# Development by Design in Colombia: Making Mitigation Decisions Consistent with Conservation Outcomes

**DOI:** 10.1371/journal.pone.0081831

**Published:** 2013-12-05

**Authors:** Shirley Saenz, Tomas Walschburger, Juan Carlos González, Jorge León, Bruce McKenney, Joseph Kiesecker

**Affiliations:** 1 Northern Andes Program, The Nature Conservancy, Bogota, Colombia; 2 Northern Andes Program, The Nature Conservancy, Quito, Ecuador; 3 Latin America Program, The Nature Conservancy, Cartagena, Colombia; 4 Global Conservation Lands Program, The Nature Conservancy, Charlottesville, Virginia, United States of America; 5 Global Conservation Lands Program, The Nature Conservancy, Fort Collins, Colorado, United States of America; University of Kent, United Kingdom

## Abstract

Mitigation policy and regulatory frameworks are consistent in their strong support for the mitigation hierarchy of: (1) avoiding impacts, (2) minimizing impacts, and then (3) offsetting/compensating for residual impacts. While mitigation frameworks require developers to avoid, minimize and restore biodiversity on-site before considering an offset for residual impacts, there is a lack of quantitative guidance for this decision-making process. What are the criteria for requiring impacts be avoided altogether? Here we examine how conservation planning can guide the application of the mitigation hierarchy to address this issue. In support of the Colombian government's aim to improve siting and mitigation practices for planned development, we examined five pilot projects in landscapes expected to experience significant increases in mining, petroleum and/or infrastructure development. By blending landscape-level conservation planning with application of the mitigation hierarchy, we can proactively identify where proposed development and conservation priorities would be in conflict and where impacts should be avoided. The approach we outline here has been adopted by the Colombian Ministry of Environment and Sustainable Development to guide licensing decisions, avoid piecemeal licensing, and promote mitigation decisions that maintain landscape condition.

## Introduction

The world is transforming rapidly, with world population projected to grow to over 9 billion by 2050 and increasing demands for food, water, energy, minerals, and other resources [1]. Over the next two decades, energy and mining companies will invest unprecedented sums – well over 20 trillion dollars – in projects around the world, from China to Colombia [1], [2]. Such projects can pose a significant challenge for biodiversity conservation, especially because in most cases the environmental mitigation process for addressing biodiversity impacts is piecemeal, opaque, and inadequate for delivering effective conservation outcomes [3]–[6].

Mitigation offers the opportunity to address the impacts of development through application of the mitigation hierarchy: avoid, minimize, restore and offset [3], [7]. But there are many problems with how mitigation is applied [3]–[6], [8]. Traditionally, mitigation has been carried out on a project-by-project basis; specific measures are implemented to mitigate project impacts at a site, usually on or adjacent to the impact site [9]. Applying the mitigation hierarchy on a project-by-project basis, often at small spatial extents, underestimates the cumulative impacts of multiple current or future development projects and undermines the hierarchy's purpose and utility [10]. Thus existing mitigation measures do not address cumulative impacts associated with development; do not provide a structured decision-making framework to determine when projects can proceed or should be avoided; and do not harness the full potential of offsets (conservation actions applied away from the development site).

### Blending systematic conservation planning and mitigation decision making

Landscape-level conservation planning is the process of locating, configuring and managing areas to maintain viability of biodiversity and other natural features [11], [12]. A conservation portfolio ( =  priority sites), the end product of conservation planning, is a set of areas selected to represents the full distribution and diversity of these features [13]. Often plans utilize an optimization approach automated with spatial analysis tools such as Marxan [14], where the portfolio is designed to meet the minimum viability needs of each biological target in a configuration that minimizes the amount of area selected [14], [15]. The key feature of a conservation plan is the clear articulation of a biodiversity vision that incorporates the full range of biological features, how they are currently distributed, and the minimum needs of each feature to maintain long-term persistence [16]–[18].

Landscape-level conservation plans can be used to guide the application of the mitigation hierarchy [10], [19]. Where plans have already been completed, proposed developments can be mapped and assessed relative to the conservation portfolio and areas overlapping could trigger the avoidance of development impacts. Alternatively, overlap between the portfolio and proposed development may result in a “re-drawing” of the conservation portfolio to re-capture habitat needed to meet the minimal needs of the biodiversity features impacted by development. If minimum viability needs cannot be met elsewhere within the study-area, the proposed development would need to minimize impacts to the degree that maintains biodiversity values or avoid impacts altogether [10]. If adopted, the latter would provide an opportunity to avoid conflict between potential development and areas that are critical for biodiversity and provide the structure to guide decisions regarding which step in the mitigation hierarchy should be applied in response to proposed development [9], [10], [20].

### Colombia: a case study in mitigation planning

Recognizing the need to improve its existing regulatory framework for mitigation, the Colombian government asked The Nature Conservancy in 2008 to help develop an approach to guide better decision making around siting and mitigation of future development [8]. While the existing regulatory system required development projects to avoid impacts to certain national and regional protected areas and ecosystems such as wetlands, mangroves, mountain grasslands “paramos,” the government was seeking a more comprehensive mitigation framework that considers cumulative impacts and provides for the maintenance of minimal needs for biodiversity and ecosystems services [8].

Colombia is home to significant petroleum and mineral deposits, but also harbors some of the most biologically diverse places on Earth, with some of the highest known bird, amphibian and invertebrate species diversity [21]–[23]. Not surprisingly, some energy resources and mineral deposits intersect areas of significant biological value [24]. Conservation of biodiversity in Colombia is under threat, in part, because the Colombian government has authorized exploration of about 24 million hectares of the estimated 79 million hectares still remaining in natural ecological systems within the country [8], [25]. The increase in development forecasted for Colombia may be compatible with biodiversity conservation if properly sited, but will still pose a challenge for conservation because of the large area potentially impacted and the resulting habitat loss and fragmentation. Many impacts can be mitigated or eliminated with appropriate siting and planning for development [26].

**Figure 1:**
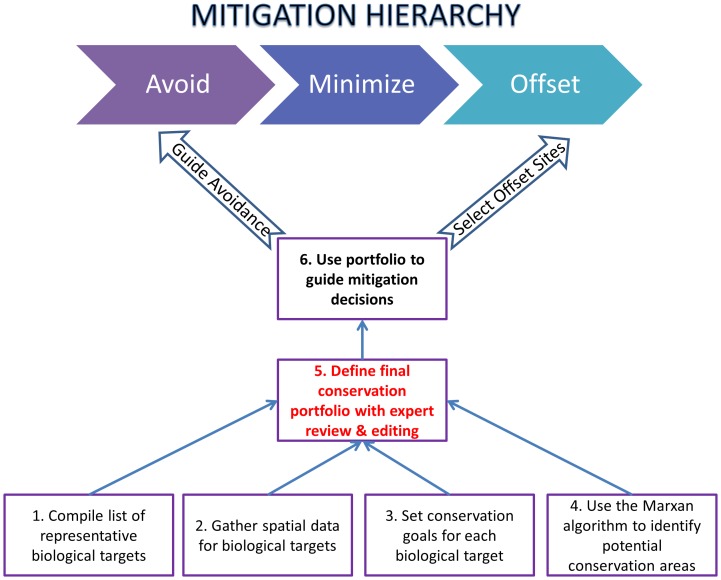
Steps used to blend landscape level conservation planning with the mitigation hierarchy, highlighting the decisions the current analysis is intended to inform: areas to avoid and the selection of offset sites.

**Figure 2:**
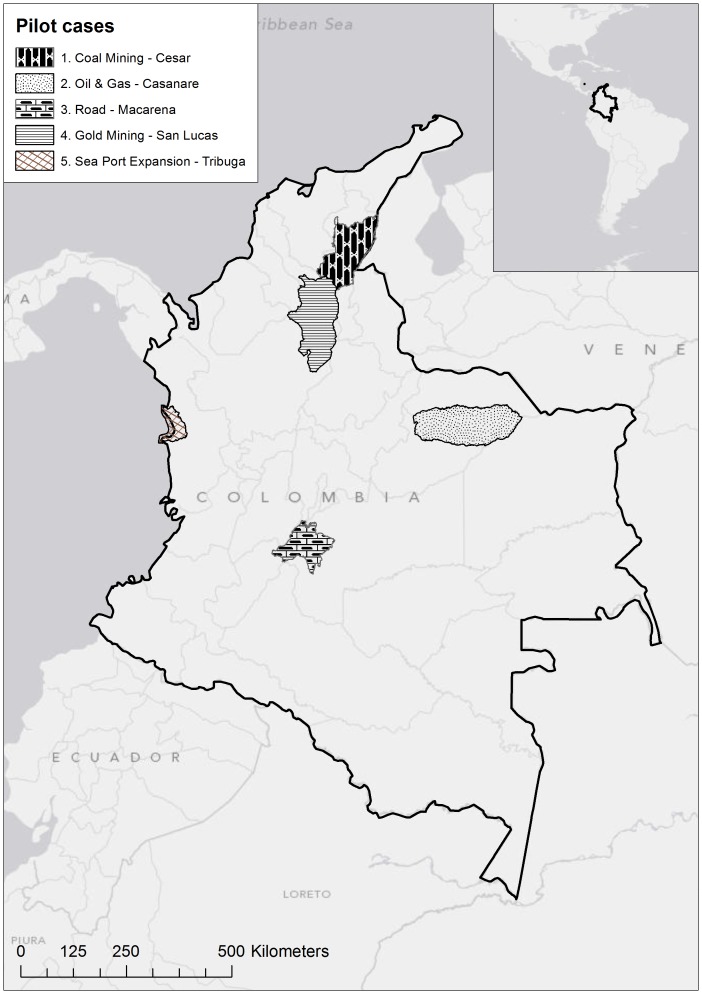
Location of development by design pilot projects within Colombia.

There is broad agreement among scholars, scientists, policymakers, and regulators that the first and most important step in the mitigation hierarchy, avoidance, is ignored more often than it is implemented [4], [5]. What are the criteria for requiring impacts be avoided altogether? Here we outline a conservation planning framework for proactively identifying future development that would be incompatible with long-term conservation goals (Figure 1). The conservation portfolios developed through this process can be used to identify areas where future development impacts should be avoided [10], [19], [20]. We focus on five landscapes where development is projected to increase rapidly over the coming years. This includes the expansion of coal mining in the Cesar River Valley, gold mining in Sur de Bolivar, highway development in Macarena, oil and gas development in Casanare and expansion of a sea port in the Bahia de Tribuga in Choco (Figure 2, Table 1). The approach we outline here has been adopted by the Colombian Ministry of Environment and Sustainable Development to guide licensing decisions, avoid piecemeal licensing, and promote mitigation decisions that maintain good landscape condition.

**Table 1:** Pilot project site descriptions.

Pilot Project	Total Area (ha)	Conservation Portfolio Area (ha) and (%) of the Project Area	Future Potential Development (ha)	Percentage of the conservation portfolio that overlaps with future development areas	Area within Conservation Portfolio that Overlaps with Future Potential Development (ha)	Percentage of Future Potential Development Area that Overlaps with Conservation Portfolio
Coal Mining in Cesar	1,285,592	509,725 (39.6%)	55,668	2.95%	15,046	27.03%
Gold Mining in Sur de Bolivar	1,668,565	1,260,600 (75.5%)	117,975	6.77%	85,308	72.31%
Port in Bahia Tribuga Choco	340,265	232,803 (68.4%)	639	0.26%	601	94.02%
Macarena Road in Meta	811,457	330,600 (40.7%)	16,712	0.86%	2,837	16.97%
Oil and Gas in Casanare	1,892,780	717,700 (37.9%)	687,367	30.57%	219,367	31.91%

## Methods

Our objective was to design an approach for identifying a conservation portfolio that, taking into account potential future development, maintains a minimum of 10 percent of the occurrence patterns of all terrestrial ecological systems in select pilot landscapes. We adopted a systematic conservation planning approach widely used to develop protected area networks that involves the following: 1) compile a list of important species and habitat types known collectively as 'biodiversity features', 2) collect spatial data on each of the biodiversity features targeted for protection, 3) set representation targets for the minimum amount of each feature intended for protection, 4) use spatial conservation prioritization software 'Marxan' in conjunction with expert based opinion to identify priority areas that meet representation targets, and 5) assess the cumulative impacts of future development and provide a regional context to better guide which step of the mitigation hierarchy should be applied (i.e. avoidance versus offsets). We sought to embed mitigation decisions into a landscape context. Previously, mitigation decisions were focused on the mining or oil and gas concessions and a small buffer around these areas defined often by the companies as part of the Environment Impact Assessments (Saenz & Walschburger unpublished data). Here we created project boundaries based on the main watersheds and administrative boundaries of municipalities within the project areas. A working group that included representatives from environmental authorities, research institutes, academia, indigenous and afro-Colombian groups, local and regional authorities and non-governmental organizations was formed to guide the process for each pilot landscape. This group helped provide the most current spatial data on our biological targets, assessments of the predictive models being developed, as well as insight into the approach being developed. We sought to apply rigorous, objective measures of conservation value whenever possible, recognizing that a quantitative assessment would require expert review and refinement.

### Compile list of representative biodiversity features

**Figure 3:**
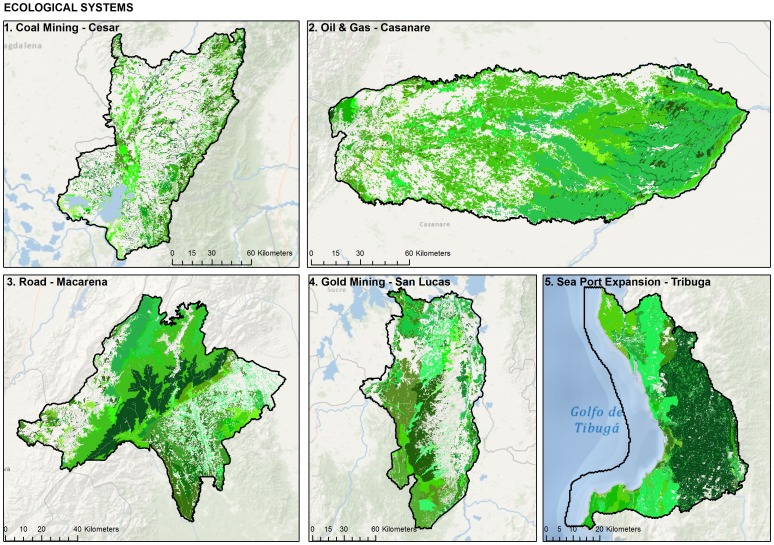
Ecological systems within each pilot project area.

**Figure 4:**
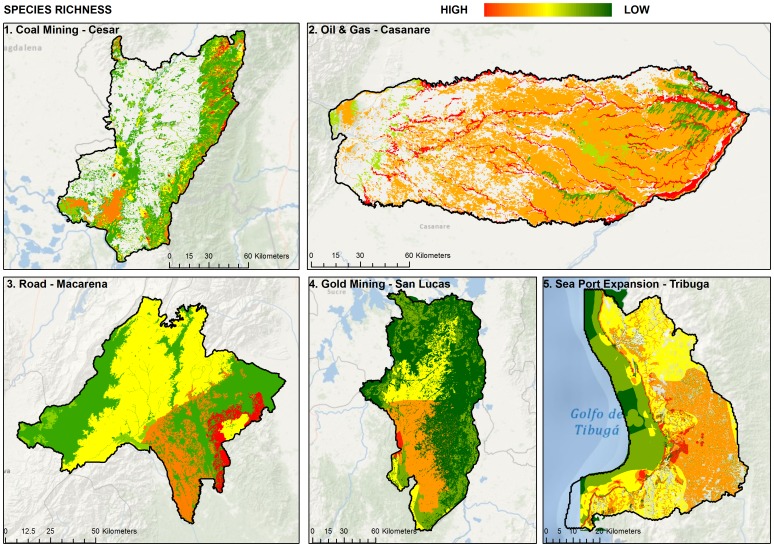
Species richness within each pilot project area.

Biodiversity cannot easily be measured completely and directly. Practitioners address this issue by selecting a set of biodiversity components that adequately represent the range of biological phenomena in the project area and can be measured effectively given existing resources. We selected a set of focal targets with sufficient breadth and depth using the “coarse-filter/fine-filter” approach consistent with The Nature Conservancy's Ecoregional Planning approach [27]. *Coarse filter* generally refers to “ecosystems”; in a more practical sense, it refers to mapped units of vegetation (Figure 3). The basic idea is that conserving a sample of each distinct vegetation type, in sufficient abundance and distribution, is an efficient way to conserve the majority of biological phenomena in the target area [28]. *Fine filter* generally refers to individual species, with specific habitat requirements or environmental relationships that are not adequately captured by the coarse filters (Figure 4). Narrow endemics and extreme habitat specialists, species with restrictive life histories, or those species that have experienced significant loss of habitat and/or are particularly sensitive to human perturbations fall into this category (i.e. IUCN Red List species). For our case studies we generated a list of biodiversity features using the coarse filter, fine filter criteria [29]–[33]. Details on targets used in each case study can be found in Table 2 and Table S1.

**Table 2:** Details of conservation portfolio design process using Marxan analysis for each of the 5 pilot landscapes.

PILOT PROJECT	PLANNING UNIT (Hexagon ha)	TOTAL NUMBER OF HEXAGONS	BOUNDARY LENGHT MODIFIER	NUMBER OF RUNS
Coal Mining in Cesar	25	490,327	0.2	500
Gold Mining in Sur de Bolivar	300	5,846	1.750	100
Port expansion in Bahia Tribuga Choco	20	16,527	1.0	100
Macarena Road in Meta	100	8,510	10	500
Oil and Gas in Casanare	100	19,424	10	500

### Spatial data for biodiversity features

We used a combination of point survey data, vegetation cover estimations and predictive model estimations to represent the spatial distribution of selected targets. For all projects we utilized the national landcover data set with maps produced at the 1∶500,000 scale [34]. At the pilot landscape level we refined this product to capture the location and delineation of each ecosystem unit with a remotely sensed exercise focused on each of the five pilot areas (Figure 3). For identification of ecosystems within each pilot landscape, we relied on a 1∶100,000 scale land cover map generate with imagery from remote sensing sensors ASTER, and ETM + Landsat, taken between 2000 and 2008. The maps were obtained through the Landsat and TerraLook collections held in the USGS archive by using USGS Global Visualization Viewer -GLOVIS-, and remote sensing sensor Cbers2, obtained from Instituto Nacional de Pesquisas Espaciais (INPE) archive [29]–[33]. The remotely sensed data along with the national landcover map were used to generate a preliminary map of ecosystems units [29]–[33]. Where available we also utilized ecoregional assessments conducted by Galindo et al. [35], [36].

Con este producto se genero un taller de expertos en el que se discutió la leyenda del mapa de ecosistemas, la delimitación y ubicación geográfica de cada una de las unidades y su pertinencia como objeto de conservación de filtro grueso.To select fine filter species targets we started with base species maps produced by NatureServe [37]. We also reviewed ecoregional assessments that were available for all of the five project areas and used species lists generated from these analyses [29]–[33], [35], [36]. Where existing species models were not available we settled on a simple approach of using deductive models by identifying the habitat preferences for each species creating binary models of suitable habitat through a series of GIS overlays based on: slope, aspect, topographic roughness, elevation (DEM), and vegetation type. The resulting species distribution models were subsequently validated by local experts (Figure 4).

Algunos criterios utilizados para la definición de la distribución potencial de las especies están basados en: rangos altitudinales, áreas geográficas descritas (municipios, departamentos, cuencas, toponimia, etc.), cartografía base y los mapas de Nature Serve (2007), donde para algunos casos de anfibios, aves y mamíferos fue de mucha utilidad

### Setting conservation goals for biodiversity features

For each project we aimed to develop a portfolio of areas that would maintain Las metas de conservación para ecosistemas o filtro grueso se establecieron tomando como referencia el mínimo de 10% para ambientes terrestres según UICN. viability of ecosystems. Based on goals adopted by Colombian government we sought to maintain a minimum of 10 percent of the occurrence patterns of all terrestrial ecological systems [38].Para el caso de ecosistemas estratégicos (bosques de galería, humedales, paramos, cuerpos de agua) se definió una meta de conservación del 100% dado el régimen de manejo y su importancia por prestación de servicios ecosistémicos.Para el caso de ecosistemas estratégicos (bosques de galería, humedales, paramos, cuerpos de agua) se definió una meta de conservación del 100% dado el régimen de manejo y su importancia por prestación de servicios ecosistémicos. Given the importance of ecosystem services provided by some ecological systems (riparian forests, wetlands, moors, bodies of water), we sought to conserve 100 percent of these systems. To set goals for ecological systems we utilized an approach developed by Pressey and Taffs [39] in which the amount of the current distribution needed to be conserved is calculated as a function of the goal as well as a function of the current distribution and landscape condition. Thus for each ecological system we calculated a distribution and condition metric.La ecuación propuesta para la estimación de la meta de conservación para cada uno de los objetos de conservación de filtro grueso fue la siguiente: The proposed equation for estimating the conservation goal for each of the terrestrial ecological systems was as follows (Table 3 shows an example of this calculation):

**Table 3:** Example of conservation goal calculation for representative ecological system.

INDICATOR	ATTRIBUTE	WEIGHT (5-1)	SCORE	TOTAL WEIGHT
	Location in biome	5-1	5	
DISTRIBUTION	Rarity	5-1	4	4.3
	Location in watershed	5-1	4	
	Nearest neighbor distance	5-1	3	
CONDITION	Average proximity index	5-1	5	3.3
	Weighted Index Form	5-1	2	

Example from San Lucas Orobiome Gold Mining Pilot for Erosional Mountain Forest Ecological System.

GOAL  =  [(10% * Distribution) + (5% * Condition)] * 1.5

GOAL  =  [(10% * 4.3) + (5% * 3.3)] * 1.5

GOAL = 90%

Conservation Goal  =  [(10% * Distribution) + (5% * Condition)] * 1.5

Goals were lower for widely distributed ecological systems compared to rarer systems. In addition goals were higher for ecological systems with lower condition relative to those with higher condition. Condition was based on a human disturbance index described below. This approach was used in three projects (coal mining in the Cesar River Valley, gold mining in Sur de Bolivar, and expansion of a sea port in Bahia Tribuga). For the remaining projects a slight modification was added following the approach used by Galindo et al. [35], [36] that incorporated future threat when calculating the goal. The proposed equation for estimating the conservation goal for each of the terrestrial ecological systems was:

Goal  =  (Rarity + Condition + Threat)

As above goals were higher for rare ecological systems and those with higher condition values. Ecological systems that are highly threatened also had a higher conservation goal. For the marine area in Tribuga, we used the conservations goals developed by Alonso et al. [40] in their marine ecoregional planning exercise for the Colombian Pacific. In all cases goals were between 35 and 100 percent and equate to the percent of the current area remaining for that ecological system. For example, a goal of 100 percent means that all of the current area remaining should be included in the conservation portfolio.

We choose not to assign specific goals to species, instead using species data to supplement the selection of sites included in the conservation portfolio. Once species models were compiled we summarized the species richness of these select target species across the pilot landscapes [37], (Figure 4). We placed the areas into categories based on Natural Jenks [41]. Details on the number of species for each category can be found in Table S1. Areas with the highest species richness were given priority for selection. All of the pilot landscapes followed this approach with the exception of the Bahia Tribuga port expansion project where we used species goals established by Alonso et al. [40].

### Selecting potential conservation areas with Marxan

To design a conservation portfolio we applied the Marxan (version 1.8.2) site-selection algorithm developed by Ball and Possingham [14]. Marxan is a siting tool for landscape conservation analysis that explicitly incorporates spatial design criteria into the site selection process. Marxan utilizes an algorithm called “simulated annealing with iterative improvement” as a heuristic method for efficiently selecting regionally representative sets of areas for biodiversity conservation [42]. Marxan allows inputs of target occurrences represented as points, polygons in a GIS environment, and allows for conservation goals to be stated in a variety of ways, such as percent area, or numbers of point occurrences etc. The program also allows for the integration of many available spatial datasets on land use pattern and conservation status, and enables a rapid evaluation of alternative configurations. The ultimate objective is to identify a portfolio of planning units that meets the goals established for each biodiversity feature at lowest cost (i.e. cost  =  condition, conservation cost in dollars, size of the reserve etc.). Marxan is not designed to act as a stand-alone reserve design solution. Its effectiveness is dependent upon the involvement of expert opinion for review and revision. Marxan is sensitive to a number of the settings that are selected by the users (i.e. Boundary Length Modifier (BLM), number of runs and iterations). In all cases we assessed the sensitivity of solution sets to different values of these variables. For example to examine how the BLM affected spatial patterns of the solution we set BLM at zero and iteratively increased it by factors of ten. In consultation with the working group we visually inspected the results and examined how variation in the BLM affected the desired degree of clustering and cost. In all cases we assessed the sensitivity of variables in consultation with the working group [43]. Information on the levels for these settings can be found in Table 2 and Table S1. For all analyses we selected hexagons (derived from a uniform grid) as the unit of analysis for running Marxan that were of sufficient spatial resolution to represent biodiversity features and also large enough to permit efficient analyses across broad landscape scales (see Table 2 & Table S1 for specific rules used for each pilot area). The effectiveness of a contiguous set of hexagon units for defining natural variability, especially among spatially heterogeneous data sets, is well documented [44]. Each hexagon was populated by summing the area of suitable habitat for the targeted ecological systems or species richness.

**Figure 5:**
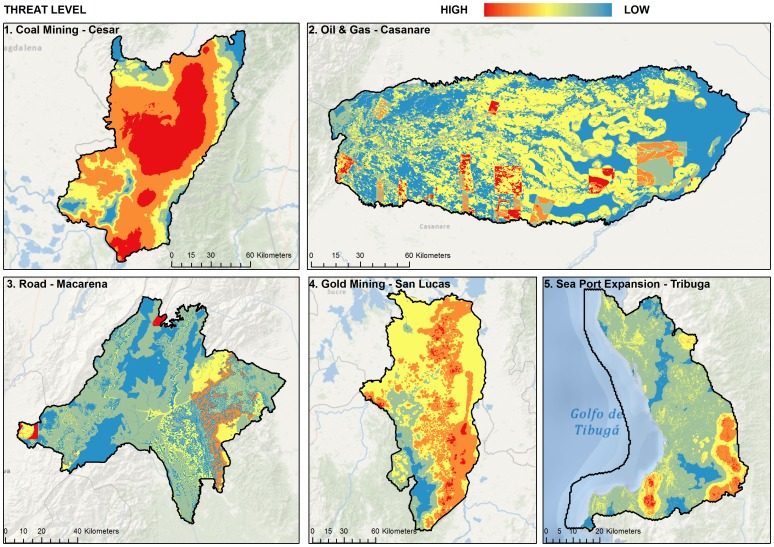
Threat Level or cost ( =  current condition) within each pilot project area.

To select conservation areas, we developed a set of decision rules. First, we guided site selection to areas with low levels of human disturbance [45]. Identification and quantification of current impacts is an integral part of ecoregional planning because the success of conservation strategies to protect species, landscapes, and ecosystems are dependent on the condition of the sites chosen [46]. The spatial characterization of the impacts is one of the determining factors in the selection of priority areas for conservation, because it directly or indirectly influences the viability of conservation of biodiversity [12], [33]. We modeled six types of impacts for the five study areas: accessibility, livestock, agriculture, mining, energy and illicit crops (for areas where applicable, Figure 5) and combined these factors to calculate an index of cumulative impacts. This impact index is a component of the “cost” function utilized by Marxan [14], [43]. As a result it was more costly to select planning units that had a high level of impact as represented by these datasets. Given the difficulty of restoration as well as uncertainty around maintaining conservation values in areas of high human influence, we felt it necessary to select areas in good condition ( =  areas with the lowest cost) and seek to keep these systems from becoming degraded rather than attempt to focus on restoration as a primary conservation action. Although, given the extensive amount of land conversion in these landscapes, it was necessary to select some degraded sites in order to meet conservation goals for some ecological systems. Next, we locked in areas [14], [43] currently listed in the countries protected areas network [29]–[33], [35], [36]. We felt that this last rule was critical given the commitment already made to conservation of these areas and the need to complement ongoing efforts to maintain biodiversity of the country.

### Using selected conservation areas to guide mitigation decisions

**Figure 6:**
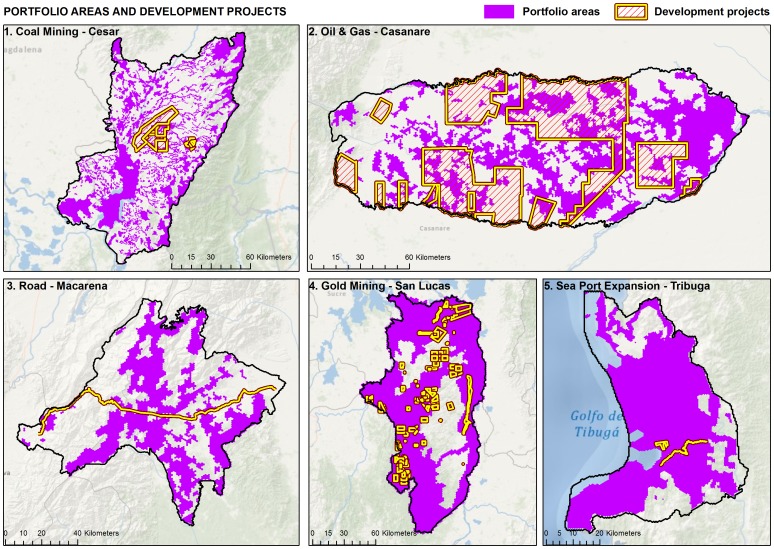
Landscape-level recommendations for the application of the mitigation hierarchy for each pilot project area. Portfolio of conservation sites selected by the ecoregional assessment in purple. Development potential outlined in yellow hash marks showing overlap between potential development and conservation priorities.

We assessed the cumulative impacts of future development projects within a regional context to better guide which step of the mitigation hierarchy should be applied (i.e. avoidance versus offsets). We examined areas selected as part of our conservation portfolios in the context of expected future development for each of the five pilot landscapes (Figure 6). We took a fairly simple and conservative approach when making decisions about which proposed developments should be avoided. Based on the amount of habitat already converted to other uses and the desire to emphasize the need for avoidance, the working group decided that any overlap between the conservation portfolio and proposed development should be avoided and permits should not be granted for these leases. By assessing how future development impacts may affect biodiversity goals in a regional context, there is an opportunity to be proactive in avoiding conflict between development and conservation goals. Decision-makers can seek solutions that support development and maintenance of natural systems.

## Results

The results of our analysis show the potential of conservation planning to proactively identify potential conflicts between conservation goals and development objectives and guide application of the mitigation hierarchy. The areas selected as part of the conservation portfolio represented from 38 percent to 75 percent of the total area of the pilot landscapes, providing some opportunity to resolve conflicts by simply re-designing development objectives to be met in areas that are not part of the conservation portfolio (Table 1, Figure 6). In general the overlap between the conservation portfolio and areas of future development represents only a small conflict for meeting conservation goals. With the exception of the gold mining activities in the Sur de Bolivar region (∼7 percent%) and the oil and gas development in the Casanare region (∼30 percent), the intersection between future development and conservation portfolio represents less than 3 percent of the conservation portfolio. However, the conflict appears more significant if we consider the proportion of the total area proposed for development that has been selected as part of the conservation portfolio. Approximately ∼17 to 94 percent of the total area proposed for development (depending on the region) overlaps with the conservation portfolio, mainly because of the smaller size of proposed development areas compared to the area needed to meet conservation goals (Figure 6, Table 1). This overlap will represent a significant challenge for development in some of the landscapes.

## Discussion

Our results illustrate how a portfolio of sites selected as part of an ecoregional assessment can be used to guide application the mitigation hierarchy identifying areas where development may and may not be compatible with landscape level conservation goals. Our results also demonstrate the flexibility that exists in applying the mitigation hierarchy when tradeoffs are examined at an appropriate landscape scale. Using a landscape scale assessment allows the flexibility to design a conservation portfolio that reduces potential conflicts between current and future potential development. By considering current impacts in the portfolio design, and incurring a higher cost for selection of sites with development potential, we steer conservation priorities away from potential conflicts where possible. Our conservation portfolio reflects these principles. Although the conservation portfolio represents 38 to 75 percent of the landscape, less than three percent of the conservation portfolio would be affected by future development activities, with two exceptions – the Casanare region (∼30 percent) and Sur de Bolivar region (∼7 percent) (Figure 6, Table 1). Moreover, the conflicts in the Casanare and Sur de Bolivar regions could be reduced through local siting and/or development approaches that minimize impacts to the degree compatible with biodiversity goals. For example, in the case of oil and gas development in the Casanare, there is significant opportunity for reducing impacts in the process of siting and designing well pads, roads and pipelines [47]. In addition, directional drilling may allow access to oil and gas resources in a manner the avoids or minimizes impacts [48].

As a proportion of the total area proposed for development the conflict with the conservation portfolio is considerable, ranging from about 17 to 94 percent depending on the region (Figure 6, Table 1). Prohibiting this proposed development could potentially result in significant economic losses. But it is important to recognize that our analysis represented development potential rather coarsely using concession boundaries, which likely overestimate the potential conflict between development and conservation. As more specific development planning information for the concessions in question becomes available, this information can be taken into account with the geospatial data available from our analysis to better inform and guide decisions. Moreover our representation of biodiversity features is relatively coarse in scale (1∶100,000 scale) and as development plans are refined it likely that biodiversity assessments will be refined as well. These refinements may make it possible to design development activities that minimizes impacts or avoids impacts to biodiversity features completely.

Given both the considerable conflict the conservation portfolio represents for development goals and the high economic value the leases likely represent, conservation planning could be adapted to further reduce conflicts with development objectives by designing a portfolio to avoid conflict with potential development [10], [19], [20]. Proposed developments can be mapped and assessed relative to the conservation portfolio and the minimum viability needs of the targets. Overlap between the portfolio and proposed development may result in a “re-drawing” of the portfolio to re-capture habitat needed to meet biodiversity goals impacted by development in areas not slated for development [10], [19], [20]. Alternatively proposed development activities can be incorporated into the cost surface incurring a higher cost for selection of sites where proposed development activities may occur, ultimately steering the selection conservation of the portfolio away from potential conflicts. Given the small overall loss the majority of these leases represent to the conservation portfolio it seems unlikely that the Colombian government will forego the revenue in favor of conservation goals. In our pilot studies we could have examined more complicated scenarios to avoid conflicts between conservation and development objectives. However, it was the decision of the working group to use the portfolio design process in a simplistic manner, using it to highlight leases that should be considered for avoidance. Future assessments seeking to either update the analysis within our focal landscapes or expand the application of this concept to other landscapes should seek to incorporate more complicated scenarios to avoid conflicts between development and conservation objectives [10], [19], [20].

Our framework attempts to address some of the key deficiencies with current mitigation practices. It provides decision makers and stakeholders the opportunity to proactively understand potential losses to biodiversity resources associated with proposed development plans, supporting informed decisions. In too many places mitigation is still carried out on a project-by-project basis, with piecemeal actions taken on-site or nearby. There is little or no consideration about how these actions contribute to wider goals for the landscape. Traditional mitigation also ignores the future. Too often mitigation is implemented without considering the projected cumulative impacts for the region from planned energy, mining, and infrastructure. More broadly, by integrating conservation and development planning at a landscape scale, it supports application of mitigation at a more appropriate ecological scale. Projecting cumulative impacts at this scale moves mitigation beyond a project-by-project approach to one that can support a dynamic vision consistent with systematic conservation planning. While we choose not to consider future potential cumulative impacts of development in our portfolio design our analysis can still help decision makers incorporate cumulative impacts into their decision making. For example a regulator considering an impact to a conservation target at one site can examine how that decision will contribute to overall potential impacts. Moreover these regulators can consider how that impact may or may not restrict the ability to meet conservation goals. If impacts represent a significant portion of the conservation goal and there is limited flexibility to meet goals elsewhere they can recommend more avoidance and minimization be utilized. The framework we present here may also be a way to improve the application of conservation planning exercises. Over the last three decades a great deal of research, funding, and effort have been put into the development of systematic conservation planning, yet these methods are used infrequently by those charged with managing landscapes [49]. By blending landscape level conservation planning with mitigation we have the potential to move these analyses out of the academic world and into the hands of the regulatory agencies responsible for the decisions that drive the majority of land-use change [10], [20].

Landscape-level plans can also improve the conservation benefits of applying the mitigation hierarchy, in particular how offsets are designed and sited [9], [10]. Most biodiversity offset legislation and policies presume “like-for-like” or “in-kind” offsets (i.e. offsets that conserve biodiversity of a similar kind to that affected by the development) [4]. At times, however, better conservation results may be obtained by placing the offset in an ecosystem of higher conservation priority [10], [50], [51]. A regional landscape perspective can provide opportunities for identifying situations where “trading up” or “out-of-kind” offsets may offer valuable alternatives. Consider, for example, development that results in impacts to a widely distributed or highly conserved target. Requiring in-kind offsets could limit the potential benefit that an offset might provide. For example, losses of a particular common habitat type could be offset in a habitat of higher priority in the region, because it is under great threat (i.e. vulnerable) or because it is the last remaining example of its kind, and is therefore irreplaceable [10], [20]. Out-of-kind offsets may also be preferable where there is an opportunity to take advantage of existing conservation management to locate the offset, or consolidating several offsets in one location.

The goal of our analyses was to illustrate a way in which gaps in the existing siting and mitigation regulatory framework for Colombia could be improved using available data and tools. The pilot sites selected to illustrate these concepts were chosen jointly by The Nature Conservancy and the Colombian Ministry of Environment and Sustainable Development (MADS) because they are expected to experience significant increases in development pressure [25]. As a result of this work MADS adopted a resolution (MADS 2010 –Resolución 1503 de 2010) and a methodology to incorporate the principles of biodiversity offsets outlined in our analyses into its licensing process for terrestrial projects [52]. For the first time, companies will be required to compensate for impacts to biodiversity in accordance with an explicit science-based framework. It will also encourage MADS to place impacts of development into a landscape perspective: highlighting the cumulative impacts of development, revealing the potential losses and making clear the need for mitigation, including both avoidance and the compensation of impacts. Prior to these changes Colombia's approach to impact mitigation focused primarily on impacts to forested systems. In some cases forest clearing resulted in the planting of fruit trees as a way to compensate for impacts (Saenz & Walschburger unpublished data). There was also a need for more structured decision-making framework to determine when projects could proceed or should be avoided. Now the MADS has guidelines for proactively evaluating the compatibility of proposed development with conservation goals and determining when impacts should be avoided [50], [52]. In addition to decisions about avoidance and minimization, the framework will support MADS in determining ecologically equivalent offset opportunities, locations where these offsets can best contribute to landscape conservation goals, and the amount of compensatory mitigation needed to address impacts [50], [51]. While only recently signed into law on August 31, 2012 and becoming effective on January1, 2013, this change in the licensing process should drive both a significant increase in, and more effective use of, funding for biodiversity conservation across Colombia [50], [51].

Despite the progress MADS's regulatory change represents, there are a number of issues that will need to be addressed to effectively implement the guidance especially as it relates to the avoidance of impacts. Since MADS's engagement in the siting and mitigation of development occurs as part of the licensing process, it may be too late in the process to require a project's impacts be avoided. In situations where direct avoidance of impacts is not possible, MADS may encourage companies to avoid impacts by calling for high rates of compensation [50], [51]. In addition, other agencies and sectors of government may have authority to make decisions regarding avoidance of impacts. For example, local municipalities may use their territorial land use planning process to guide and shape which resources are made available for development. The Colombian government has started to define a process to identify areas necessary to maintain ecological structure for the entire country, asking what is the minimum area needed to maintain biodiversity and ecosystem services [25]. The framework we have presented here could help guide that process.

## Conclusion

By avoiding or minimizing impacts to irreplaceable biodiversity features, then ensuring that damaged ecosystems are restored on site, using the best available technology, and finally offsetting any remaining residual impacts, we can provide a framework that is consistent with sustainable development. A landscape vision is essential, because it helps us to move beyond a project by project, business-as-usual approach to addressing conflicts between development and conservation goals. Given the extensive amount of development projected for Colombia, a requirement that development projects achieve no-net-loss outcomes for biodiversity could be the impetus needed to conserve biodiversity across the country.

## Supporting Information

Table S1 **Ecological systems and species selected in each pilot project.**
(DOCX)
