# Supplementary material for: Development by Design in Colombia: Making Mitigation Decisions Consistent with Conservation Outcomes
Source: PLoS One. 2013 Dec 5;8(12):e81831. doi: 10.1371/journal.pone.0081831 (PMC3855343; doi:10.1371/journal.pone.0081831)
Supplement: Table S1 — Ecological systems and species selected in each pilot project. (DOCX) [file pone.0081831.s001.docx]

**Table S1.** Details on the ecological systems and species selected in each conservation portfolio. Goals for each ecological system is included as a percent of the current distribution. The number of species is included by taxa along with species richness categories that are represented as a range of the number of species included in each category.

| **PILOT Project** | **TOTAL AREA (Ha)**  **(Details on the Conservation Portfolio)** | **Ecological systems** | **Goals %** | **SPECIES (Species Richness Categories)** |
| --- | --- | --- | --- | --- |
| Coal Mining in Cesar | 1,285,592  (For the Cesar Valley pilot landscape where expansion of coal mining is projected, the optimal portfolio consisted of 24 priority conservation areas covering a total area of 509,725 hectares. This represents about 39% of the total area of the study area. The portfolio was dominated by conservation targets associated with the Caribbean Helobioma. Of the 24 conservation areas selected about 10 consist of floodplain forests dominated by gallery (riparian) forests, representing approximately 44% of the total portfolio. In addition areas of savannas and shrublands, Scrub and dry xerophytic vegetation consist of about 19,900 ha. While the intersection between the conservation portfolio and the currently licensed mining concessions is minimal in Cesar, given the significant amount of habitat that has already been converted to other uses about five percent of the mining concessions, those overlapping with the conservation portfolio, would be expected to have impacts avoided [41].) | Riparian lake | 100 | **Mammals (17):** Callicebus torquatus, Leopardus tigrinus pardinoides, Lontra longicaudis, Myrmecophaga tridactyla Arteta, Panthera onca centralis, Saguinus oedipus, Tremarctos ornatus, Trichechus manatus , Ateles hybridus hybridus, Tapirus terrestris terrestris, Puma concolor concolor, Mazama americana, Bradypus variegatus, Alouatta seniculus, Cabassous centralis, Leopardus pardalis, Leopardus wiedii. **Birds (23):** Pauxi pauxi, Anthocephala floriceps floriceps, Ara militaris, Basileuterus conspicillatus, Campylopterus phainopeplus, Capito hypoleucus, Chauna chavaria, Clytoctantes alixii, Crax alberti, Dendroica cerulea, Metallura iracunda, Odontophorus atrifrons, Ognorhynchus icterotis, Oroaetus isidori, Gypopsitta pyrilia, Schizoeaca perijana, Vultur gryphus, Vermivora chrysoptera, Pyrrhura pantchenko, Ortalis garrula, Chlorostilbon gibsoni, Picumnus cinnamomeus, Synallaxis candei. **Reptiles (3):** Crocodylus acutus, Geochelone carbonaria, Podocnemis lewyana. **Amphibians (3):** Centrolene tayrona, Eleutherodactylus cuentasi, Cryptobatrachus boulengeri. **fishes (10):** Abramites eques, Ageneiosus pardalis, Brycon moorei, Curimata mivartii, Ichthyoelephas longirostris, Plagioscion magdalenae, Prochilodus magdalenae, Pseudouplatystoma magdaleniatum, Salminus affinis, Sorubim cuspicaudus. **Plants (25)**: Acrocomia aculeata, Astrocaryum malybo, Aspidosperma polyneuron, Brosimum alicastrum, Bulnesia arbórea, Cedrela odorata, Elaeis oleífera, Guaiacum officinale, Podocarpus oleifolius, Aspidosperma megalocarpon, Cedrela fissilis, Haematoxylum brasiletto, Pradosia colombiana, Parinari pachyphylla, Swietenia macrophylla, Sabal mauritiiformis, Espelatia periajaensis, Libanothamnus divisoriensis, Puya grantii, Hypericum baccharoides, Pentacalia perijaensis, Aragoa romeroi, Belencita nemorosa, Chaetolepis perijaensis (1-4, 5-8, 9-12, 13-16, 17-27) |
|  |  | Aquatic vegetation | 100 |  |
|  |  | Swamp forest | 100 |  |
|  |  | Riparian forest and shrubland | 76 |  |
|  |  | Grassland riparian forest | 100 |  |
|  |  | Shrub grassland (en lomerio ondulo) | 81 |  |
|  |  | Shrub grassland (en lomerio quebrado) | 80 |  |
|  |  | Shrub grassland in foothills | 93 |  |
|  |  | Dry savanna | 83 |  |
|  |  | Semi dense dry forest in lomerio | 79 |  |
|  |  | Semi dense dry forest in mountain | 80 |  |
|  |  | Riparian dry forest | 100 |  |
|  |  | Semi dense sub-andean mountain forest | 100 |  |
|  |  | Dense sub-andean mountain forest | 84 |  |
|  |  | Riparian sub-andean mountain forest | 67 |  |
|  |  | Dense Andean mountain forest | 92 |  |
|  |  | Riparian andean mountain forest | 62 |  |
|  |  | Mountain grasslands (paramos) | 78 |  |
| Gold Mining in Sur de Bolivar | 1,668.565  (The portfolio developed for the of Southern Bolívar - San Lucas landscape where gold mining is expanding is composed of nine priority areas. The final portfolio covers an area of 1,260,600 ha, of which 42%. are areas already disturbed and should be considered for possible restoration programs.) | Riparian lake (bogs) | 100 | **Mammals (12):** Ateles hybridus brunneus, Saguinus leucopus, Tapirus terrestris, Tremarctos ornatus. **Birds (10):** Ara militaris,Capito hypoleucus carrikeri, Cercomacra parkeri, Chauna chavaria, Clytoctantes alixi, Crax alberti, Habia gutturalis, Melanerpes pulcher Phylloscartes lanyoni, Pionopsitta pyrilia. **Reptiles (2)**:Geochelone carbonaria, Podocnemis lewyana. **Fishes (11)**: Abramites eques, Ageneiosus caucanus, Cochilodon hondae, Curimata mivartii, Salminus affinis, Sorubim lima. **Plants (24):** Aniba perutilis Cariniana pryryformis, Caryocar amydaliferum, Cedrela odorata Guaiacum officinale, Isidodendron tripterocarpum Juglans neotropica, Pachira quinata, Prioria copaifera Quercus humboldtii, Swietenia macrophylla, Clatrotropis brunnea Peltogyne paniculata, Dipterix oleifera, Catostema digitata, Gustavia romeroi, Lecythis tuyrana, Lecythis mesophylla, Licania platypus Caryocar glabrum, Vascivaea podocarpa, Astrocaryum malibo Elais oleifera, Brosimum alicastrum. (1-4, 5-9, 11-14, 14-20, 20-26) |
|  |  | Flood plain forest | 43 |  |
|  |  | Alluvial valley forest | 44 |  |
|  |  | Wetlands | 100 |  |
|  |  | swamp vegetation | 37 |  |
|  |  | Alluvial plain savannas | 100 |  |
|  |  | Forest in structural and erosional mountain | 57 |  |
|  |  | Mountain forest in warm weather fluiogravitational | 46 |  |
|  |  | Mountain forest in moist warm fluiogravitational weather | 52 |  |
|  |  | Mountain forest in temperate wet fluiogravitational | 56 |  |
|  |  | Mountain forest in temperate fluiogravitational very wet | 58 |  |
|  |  | Mountain forest in a temperate climate fluiogravitational | 100 |  |
|  |  | Acuatic vegetation in mountain | 47 |  |
|  |  | Savannas vegetation | 66 |  |
|  |  | In fluviogravitational hills forest | 45 |  |
|  |  | Forest in structural and erosional hills | 45 |  |
|  |  | Aquatic vegetation | 68 |  |
|  |  | Forest in Lomerio in mountain forest | 40 |  |
|  |  | Dry forest in hills | 43 |  |
|  |  | dry forest in mountain | 43 |  |
|  |  | Foothill forest in dry forest wetland biome | 52 |  |
|  |  | Aquatic vegetation in dry forest | 100 |  |
|  |  | Savanna in dry forest biome | 100 |  |
| Port in Bahia Tribuga Choco | 343,265  (For the Tribuga Choco port project the final portfolio covers an area of 232,803hectares, of which 14% are areas currently disturbed and should be considered for possible restoration programs. Despite the reliance on disturbed areas the portfolio was able to meet the goals set for all ecosystems and species. The portfolio focused on selecting the coarse filter objects of marsh vegetation along riparian areas and lower terraces of rivers. | Estuarine Forest | 100 | **Species (18):** Bird communities of the tropical rainforest and mangroves, Community amphibians, Panthera onca, Puma concolor, Community mammalian prey, terrestrial turtle, Community psittácidos sharks, megaptera, deep-water shrimp, shallow-water shrimp, dolphins tursinus, hake, turtle nesting beaches, groupers and grouper, other bony fish, Breeding birds. (Not Applicable) |
|  |  | forest in Monocline Crestones dissected | 47 |  |
|  |  | forest in Monocline and Composite Spine Anticline | 45 |  |
|  |  | Halobiome forest | 30 |  |
|  |  | Riparian forest | 42 |  |
|  |  | estuarine forest in grasslands | 100 |  |
|  |  | forests on slopes and hills | 45 |  |
|  |  | forest in erosional mountains | 47 |  |
|  |  | forest in branched mountains | 33 |  |
|  |  | forest in mountains and hills branches | 48 |  |
|  |  | forest in aluvio-coluvial foothills | 45 |  |
|  |  | forest in beach | 100 |  |
|  |  | Littoral beaches | 10 |  |
|  |  | Wetlands | 30 |  |
|  |  | soft bottoms | 30 |  |
|  |  | coral formations | 100 |  |
|  |  | mangroves | 100 |  |
|  |  | beaches | 30 |  |
|  |  | secondary vegetation | 100 |  |
|  |  | rocky Coastlines | 30 |  |
|  |  | freshwater systems | 50 |  |
|  |  | Riscales | 100 |  |
| Macarena Road in Meta | 811,457  (For the Macarena Road expansion the final portfolio covers an area of 330,600 hectares and consisted of 18 priority areas, of which ~5% are areas currently disturbed and in need of restoration. This included the special management area of “La Macarena” Protected Area. The portfolio focused on selecting Buffer Zone Forests and Forest peaks of the Sierra de Gigante. In addition the portfolio focused on forested areas in the High River Preservation Zone of Guayabero and Piedmont Riparian Forests in the northern portion of the “La Macarena” area.) | Humid tropical rainforest in hills and hillsides | 70 | Mammals (15sp. ): Aotus griseimembra, Aotus lemurinus, Ateles belzebuth belzebuth, Callicebus cupreus ornatus, Cuniculus taczanowskii, Dynomis branickii, Lagothrix lagothricha lugens, Leopardus tigrinus pardinoides, Leopardus wiedii, Marmosops fuscatus, Myrmecophaga tridactyla, Panthera onca, Priodontes maximus, Pteronura brasiliensis, Tapirus pinchaque, Tapirus terrestris, Tremarctos ornatus. Birds (24 sp.): Aburria aburri, Aburria pipile (Pipile pilpile), Anhima cornuta, Ara macao, Ara severus, Cissopis leverianus, Crax alector, Dendroica cerúlea, Gallinula melanops, Grallaria alleni, Gypopsitta pyrila (Syn. Pionopsitta pyrilia), Harpia harpyja, Hypopyrrhus pyrohypogaster, Mitu tomentosa, Morphnus guianensis, Patagioenas fasciata (Syn. Columba fasciata), Rupicola peruvianus, Sporophila plúmbea, Touit stictopterus, Vermivora chrysoptera, Wilsonia Canadensis. Reptiles (5sp.): Crocodilus intermedius, Geochelone carbonaria, Geochelone denticulata, Podocnemis expansa, Podocnemis unifilis. Amphibians (10sp. ): Atelopus guitarraensis, Bolitoglossa altamazonica, Ceratophrys cornuta, Cochranella adiazeta, Dendrophryniscus minutus, Gastrotheca nicefori, Hemiphractus johnsoni, Phyllomedusa tarsius, Pristimantis savage, Rahebo glaberrimus. Fishes (10 sp.):Apteronotus macrostomus, Brycon amazonicus, Curimata mivartii, Ichthyoelephas longirostris, Prochilodus magdalenae, Pseudoplatystoma fasciatum, Salminus affinis, Salminus hilarii, Sorubim cuspicaudus, Trychomycterus migrans. Plants (33 sp.): Alzatea verticillata, Aniba perutilis, Aspidosperma polyneuron, Attalea insignis, Axonopus morronei, Bactris gasipaes var. Chichagui, Billia rosea, Cattleya trianae, Cedrela odorata, Ceroxylon alpinum, Ceroxylon vogelianum, Eschweilera cabrerana, Espeletia cabrerensis, Heliconia marginata, Hyptis melissoides, Iriartea deltoidea, Juglans neotropica, Minquartia guianensis, Myrocarpus venezuelensis, Pachira quinata, Passiflora arbórea, Passiflora tolimana, Pitcairnia arenícola, Pitcairnia tolimensis, Podocarpus oleifolius, Pterocarpus officinalis, Quercus humboldtii, Schizolobium parahybum, Scutellaria parrae, Syagrus sancona, Terminalia amazonia. (1-4, 5-10, 11-15, 15-20, 21-32) |
|  |  | Humid Tropical rainforest in erosional mountains | 58 |  |
|  |  | Humid Tropical rainforest on alluvial fans and terraces | 57 |  |
|  |  | Humid tropical rainforest in fluvio-erosional mountains | 39 |  |
|  |  | Pluvial tropical rainforest in fluvio-erosional mountains | 44 |  |
|  |  | Humid tropical Rainforests in alluvial terraces | 58 |  |
|  |  | Humid to pluvial tropical rainforest on hills and low hillsides | 73 |  |
|  |  | Pluvial tropical rainforest in fluvio-erosional mountains | 55 |  |
|  |  | Pluvial tropical rainforest in intra-mountain alluvial valleys | 65 |  |
|  |  | Humid tropical in non-dissected alluvial fans | 71 |  |
|  |  | Humid tropical rainforest on alluvial plains of meandric rivers | 54 |  |
|  |  | Xerophitic vegetation and shrubland on sandstone | 56 |  |
|  |  | Xerophitic vegetation and shrubland on fluvio-erosional mountain | 55 |  |
|  |  | Xerophitic vegetation and shrubland on non-dissected alluvial fans | 65 |  |
|  |  | Subandean pluvial forests in structural hillsides | 58 |  |
|  |  | Subandean humid forests in hills and mountains of the Sierra de la Macarena | 42 |  |
|  |  | Subandean Humid Forests in structural hillsides | 67 |  |
|  |  | Sub-andean Humid Forests in fluvio-erosional mountains | 70 |  |
|  |  | Andean humid and pluvial forests in fluvio erosional mountains | 45 |  |
|  |  | Highland humid grasslands (páramos) in glacial mountains | 45 |  |
|  |  | Humid forests and subparamos (grasslands) in glacial mountains | 59 |  |
| Oil and Gas in Casanare | 1,892,780  (For the hydrocarbon development in Casanare the portfolio consists of 24 priority areas. Within the portfolio of priority areas, “Zamaricote Hill” forest and the forest of Motuz watershed dominated the areas selected. The portfolio also highlighted the areas of riparian forest along the Rio Meta as well as a complex of savannas and forests like “Los Morichales” and “El Encanto”.) | High Dense Rainforest in structural-erosional hills | 34 | Birds (23 sp.): Anas cyanoptera, Anas discors, Anhima cornuta, Anthus lutescens, Ara severa, Aratinga acuticauda, Basileuterus cinereicollis, Cacicus uropygialis, Cercibis oxycerca, Ciconia maguari, Cissopis leverianus, Cranioleuca vulpina, Crax alector, Mitu tormentosum, Myrmotherula cherriei, Neochen jubata, Phacellodomus rufifrons, Pionopsitta pyrilia, Polystictus pectoralis, Sporophila plumbea, Vermivora chrysoptera, Seiurus noveboracensis, Sayornis nigricans, Jabiru mycteria Reptiles (5): Crocodilus intermedius, Podocnemis unifilis, Podocnemis expansa, Podocnemis vogli, Geochelene carbonaria Amphibians (10): Dendropsophus mathiassoni, Pseudopaludicola llanera, Scinax wandae, Scarthyla vigilans, Pipa pipa, Dendrophryniscus minutes, Rhaebo glaberrimus, Physalaemus fischeri, Pristimantis medemi, Bolitoglossa altaamazonica Fishes(26 sp ):Aequidens metae, Apteronotus galvisi, Apteronotus_macrostomus, Astyanax integer, Bryconamericus alpha, Bryconamericus cismontanus, Bryconamericus cristiani, Bryconamericus loisae, Bujurquina mariae, Cetopsis orinoco, Charax metae, Creagrutus bolivari, Farlowella vittata, Hemigrammus barrigonae, Lasiancistrus tentaculatus, , Mikrogeophagus ramirezi, Moenkhausia metae, Orinocodoras eigenmanni, ,Oxydoras sifontesi, Parodon apolinari, Prochilodus mariae, Pyrrhulina lugubris, Semaprochilodus laticeps, Trychomycterus_dorsostriatus, Trychomycterus_migrans Plants (15): Piranhea trifoliata, Parahancornia oblonga, Inga gracilifolia, Hymenachne amplexicaulis, Fissicalyx fendleri (Benth), Bowdichia virgilioides, Bactris major (Jacq.), Bactris guineensis, Attalea insignis, Andropogon bicornis, Attalea butyracea, Acosmium nitens, Mauritia flexuosa, Carapa guianensis, Caraipa llanorum. (1-4, 5-10, 11-15, 15-20, 21-32) |
|  |  | High Dense Rainforest in structural –erosional mountain | 39 |  |
|  |  | High Dense Rainforest in fluvio-gravitational mountain | 47 |  |
|  |  | High Dense Rainforest in antique tectonized foothill | 49 |  |
|  |  | High Dense Rainforest in alluvial valleys | 46 |  |
|  |  | Medium dense Rainforest in dilluvial-alluvial foothill | 50 |  |
|  |  | Medium dense Rainforest in antique foothill | 53 |  |
|  |  | Medium dense Rainforest on alluvial terraces of Andean rivers | 43 |  |
|  |  | Medium dense Rainforest on low terraces of Andean rivers with eolic influence | 59 |  |
|  |  | Forested savanna in low terraces with eolic influence | 54 |  |
|  |  | Savanna in dilluvial-alluvial foothill | 56 |  |
|  |  | Savanna on dunes in alluvial plains | 52 |  |
|  |  | Savanna in antique tectonized foothill | 61 |  |
|  |  | Flooded savanna in low terraces with eolic influence | 46 |  |
|  |  | Seasonal flooded savanna on high alluvial terraces of Andean rivers | 50 |  |
|  |  | High Dense Forest in floodplain of andean rivers | 42 |  |
|  |  | Medium Dense Foretst in floodplains of in alluvial plains with eolic influence | 45 |  |
|  |  | Forested savanna in floodplains of andean rivers | 62 |  |
|  |  | Flooded savanna in floodplains of andean rivers | 64 |  |
|  |  | Swamp vegetation in andean river depressions | 58 |  |
|  |  | Swamp vegetation in depressions with eolic influence | 64 |  |
